# Morphological and molecular characterization of *Acrobeloides saeedi* Siddiqi, De Ley and Khan, 1992 (Rhabditida, Cephalobidae) from India and comments on its status

**DOI:** 10.21307/jofnem-2020-027

**Published:** 2020-04-28

**Authors:** Aasha Rana, Aashaq Hussain Bhat, Suman Bhargava, Ashok Kumar Chaubey, Joaquín Abolafia

**Affiliations:** 1 Nematology Laboratory, Department of Zoology, Chaudhary Charan Singh University, Meerut-250004, India; 2 Departamento de Biología Animal, Biología Vegetal y Ecología, Universidad de Jaén, Campus Las Lagunillas, s/n, 23071 Jaén, Spain

**Keywords:** 18S rDNA, 28S rDNA, *Acrobeloides bodenheimeri*, *Acrobeloides gossypii* n. syn., *Acrobeloides ishraqi* n. syn., *Acrobeloides longiuterus*, *Acrobeloides maximus*, *Acrobeloides mushtaqi* n. syn., description, ITS rDNA, taxonomy

## Abstract

Two cultured populations of *Acrobeloides saeedi* are described from India. Morphologically and morphometrically this material agrees with other species of the *Maximus*-group (*A. bodenheimeri*, *A. longiuterus*, and *A. maximus*), especially with *A. longiuterus*. However, molecular studies based on 18 S, 28 S and ITS rDNA confirmed the Indian material is well differentiated from all of these species. According to this, *A. saeedi* is considered a valid taxon distinguished mainly from *A. bodenheimeri* by having dextral female reproductive system (vs sinistral), from *A. longiuterus* by having larger females (1.03-1.57 vs 0.57-0.88 mm) and from *A. maximus* by having seta-like labial processes (vs absent) and males as frequent as females (vs males very infrequent). Molecular and phylogenetic studies revealed the present specimens to be conspecific to undescribed *Acrobeloides* sp. population from Iran, and hence, both regarded to be conspecific to each other. In addition, other similar species are revised: *Acrobeloides ishraqi* is considered new junior synonym of *A. saeedi, Acrobeloides mushtaqi* is considered new junior synonym of *A. bodenheimeri*, while *Acrobeloides gossypia* is also considered junior synonym of *A. saeedi*.

*Acrobeloides saeedi* was described by Siddiqi et al. (1992) to erect the material previously described as *Cephalobus litoralis* (Akhtar, 1962; Andrássy, 1984) from Pakistan by Saeed et al. (1988). This last material, based only in two females was observed having morphology and morphometry somewhat different (Siddiqi *et al.*, op. cit.) with respect to the type material of *Paracephalobus litoralis* described by Akhtar (1962) from Pakistan. Later, Khan and Hussain (1997) proposed the new genus *Rafiqius* to include *A. saeedi* and other morphological related species as *A. bodenheimeri* (Steiner, 1936; Thorne, 1937). This newly proposed genus was differentiated from *Acrobeloides* (Cobb, 1924) according to the morphology of the lip region, having seta-like processes at labial primary axils. However, the creation of this new genus was considered unjustified by De Ley et al. (1999). Unfortunately, none of these studies provided molecular study.

**Figure 1:**
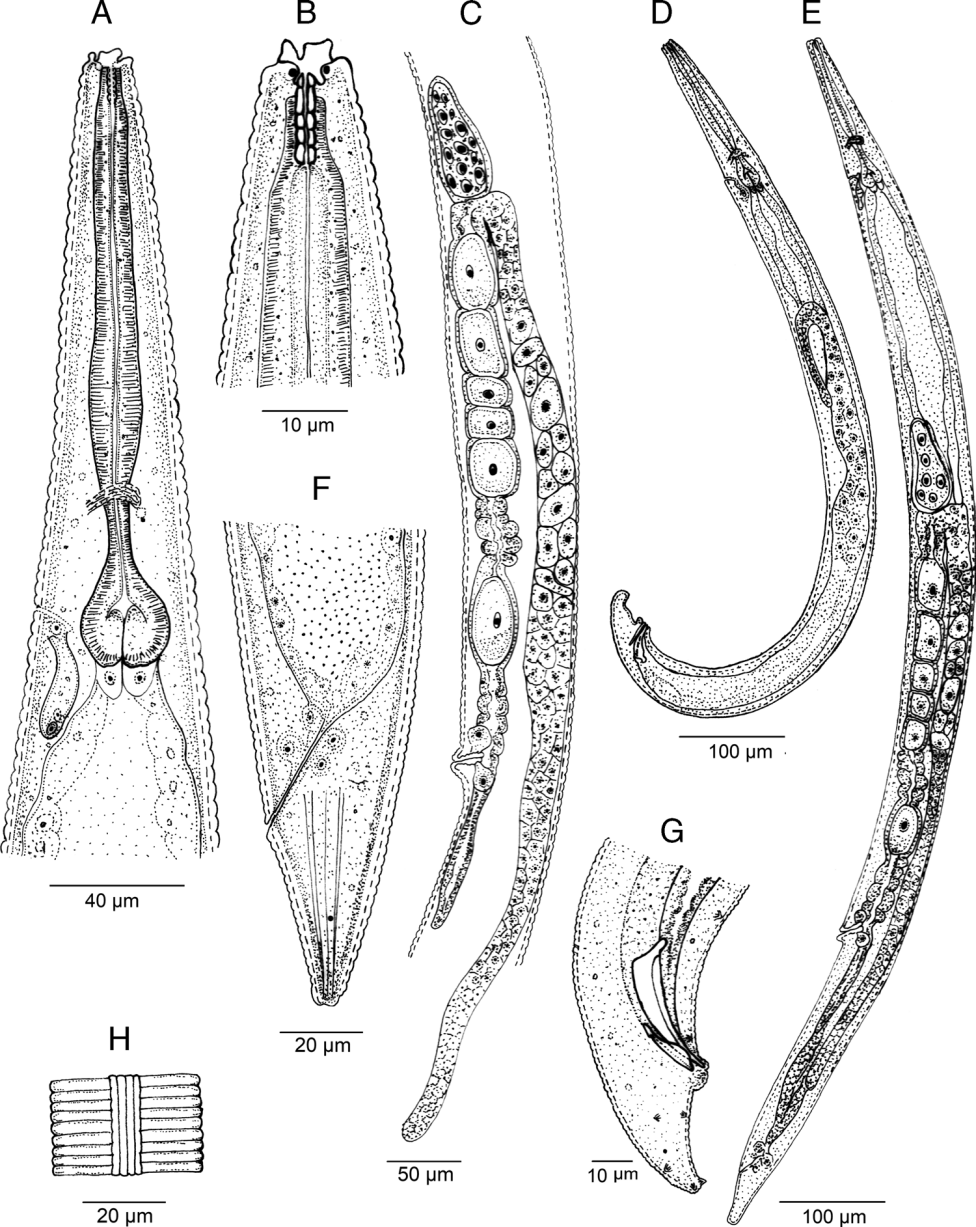
*Acrobeloides saeedi* (isolate KMW) (Siddiqi et al., 1992) (line drawing). A: adult neck region; B: anterior end; C: female reproductive system; D: entire male; E: entire female; F: female posterior end; G: male posterior end; H: lateral field.

**Figure 2:**
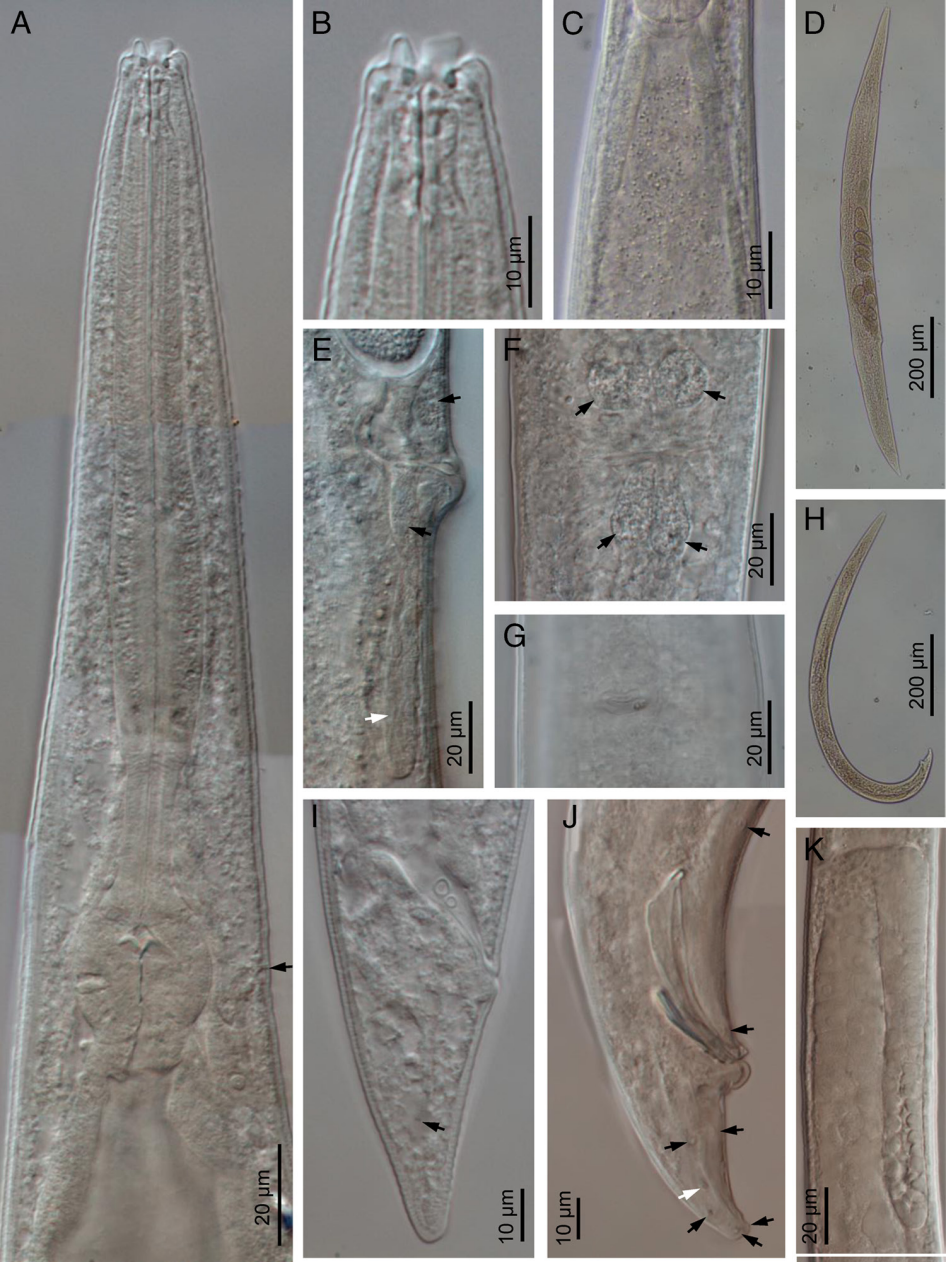
*Acrobeloides saeedi* (Siddiqi et al., 1992) (light microscopy). A: neck (arrow pointing the excretory pore); B: stoma; C: intestinal cardiac part with bacteria; D: entire female; E, F: vagina region in lateral and ventral views, respectively (black arrows pointing the vaginal glands, white arrow pointing the postvulval uterine sac); G: vulva; H: entire male; I: female posterior end; J: male posterior end; K: testis.

**Figure 3:**
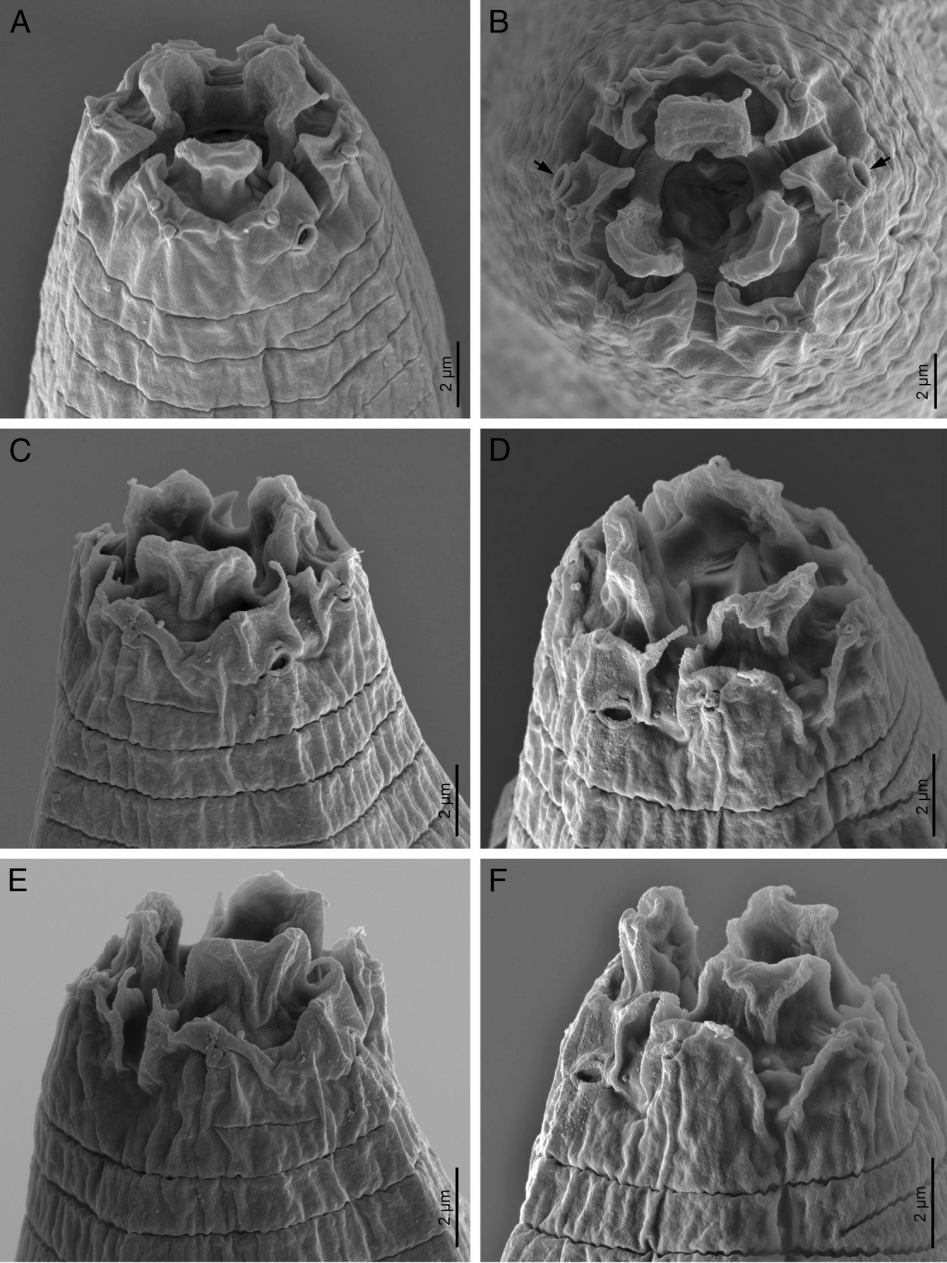
*Acrobeloides saeedi* (Siddiqi et al., 1992) (scanning electron microscopy). A-B: male lip region (arrows pointing the amphids); C-F: female lip region.

**Figure 4:**
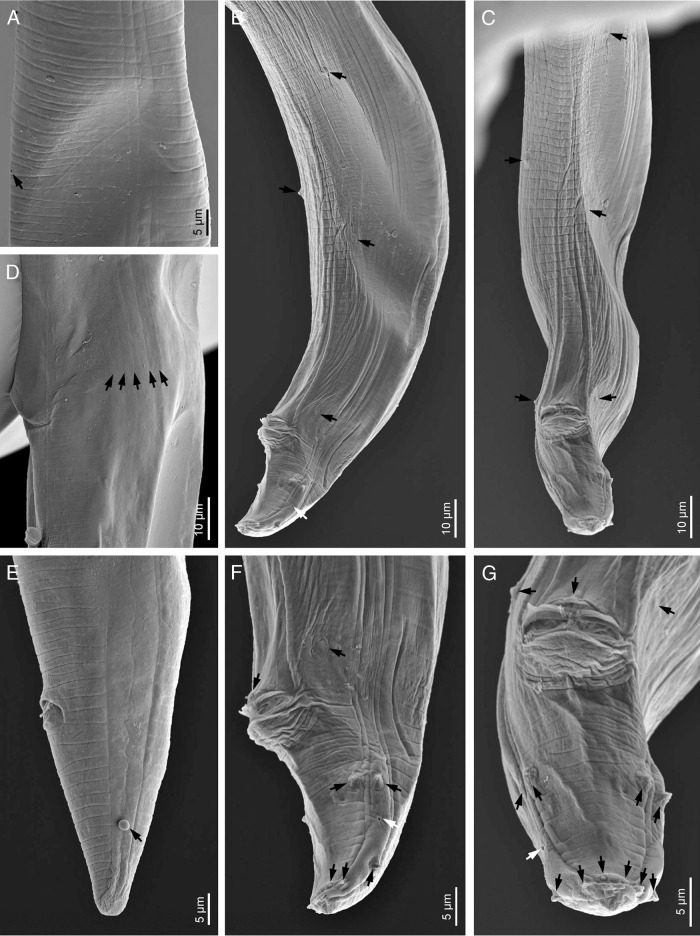
*Acrobeloides saeedi* (Siddiqi et al., 1992) (scanning electron microscopy). A: cuticle at excretory pore level (arrow); B, C, F, G: male posterior end in left lateral (B, F) and ventral (C, G) views (black arrows pointing the genital papillae, white arrows pointing the phasmids); D: lateral fields (arrows pointing the longitudinal incisures); E: female posterior end (arrow pointing the phasmid).

With respect to the isolation of soil nematodes using the *Galleria* soil baiting technique of Bedding and Akhurst (1975), the insect associate nature of some *Acrobeloides* species has been previously reported (Azizoglu et al., 2016). Besides their insect associate nature, their infestation has also been observed with some mollusks, arthropods, and annelids (Grewal et al., 2003). Kraglund and Ekelund (2002) reported infestation of *A. nanus* (de Man, 1880; Anderson, 1968) in earthworm cocoons. Baquiran et al. (2013) studied the association of these nematodes with microbes and repeatedly observed the presence of three bacterial species in association with *A. maximus* (Thorne, 1925, 1937). Later, Thiruchchelvan et al. (2018) found a free-living nematode similar to *A. longiuterus* (Rashid and Heyns, 1990; Siddiqi et al., 1992) in Sri Lanka infecting crop pests. Additionally, Suman et al. (2020) collected other rhabditid species, *Distolabrellus veechi* Anderson, 1983, from soil samples using the insect baiting technique. Their involvement in soil nutrient cycle and soil mineralization is well evident and during these processes, they interact with many arthropods and other invertebrate species, which may be phoretic to pathogenic, thus may be important for their use in biological control programs.

During a survey of soil nematodes in Meerut, Uttar Pradesh, India, two isolates of *Acrobeloides* were obtained and were labeled as KMW and DH1. Study of the specimens of these two populations showed that they were conspecific to *A. saeedi* (Siddiqi et al., 1992). Detailed redescription of this species based in morphological and morphometrical data is provided. We also provided a high quality photographic documentation of important morphological characters of *A. saeedi* through light microscopy (LM) and scanning electron microscopy (SEM). Additionally, molecular data of this species based in the D2-D3 region of the 28 S rDNA, 18 S rDNA, and internal transcribed spacer (ITS) regions of rDNA genes are included to support the morpho-taxometrical studies. This is the first molecular study of this species and its first valid report from India.

## Materials and methods

### Nematode isolation, culture, and processing

Soil samples were collected from agricultural farmlands in Mawana, Meerut (28°9´N, 77°71´E, and elevation of 225 m), India, and were tested for the presence of nematodes. Nematode specimens were isolated from two soil samples by *Galleria* soil baiting technique and were designated as DH1 and KMW. The cadavers were transferred to white trap (White, 1927) after proper washing with double distilled water and sterilization with 1% NaOCl. The nematodes that emerge in white trap were harvested, and stored in 250 ml tissue culture flasks in incubator at 15°C as described by Bhat et al. (2019). For observations and morphometrics, third-stage juveniles (200) were injected to larvae of *Galleria mellonella* by Insulin Syringe 1 ml and larvae were killed within 36 hr at 27°C. The dead larvae were then transferred to white trap. The adult generations and third-stage juveniles were collected from white trap which emerge into water within six to seven days. These specimens were then killed with hot water, transferred to TAF (2% triethanolamine and 7% formaldehyde) for fixation. The fixed nematodes were processed to dehydrated glycerine as described by Seinhorst (1959) and mounted in pure glycerine on permanent glass-slides (Siddiqi, 1964).

### Light microscopy (LM)

Nematode specimens were observed for morphological characters under phase contrast microscope (Nikon Eclipse 50i) and light microscope (Magnus MLX) while morphometric characters were measured with built-in software of the Nikon Eclipse 50i (Nikon DS–L1). Demanian indices (de Man, 1880) and other morphometrical ratios were calculated. Line drawings were made with the help of drawing tube attached to the Nikon microscope provided with differential interference contrast (DIC) optics. Images were taken with the Nikon microscope that was provided with DIC optics and Nikon Digital Sight DS-U1 camera. Micrographs were edited using Adobe® Photoshop® CS. The terminology used for the morphology of stoma and spicules follows the proposals by De Ley et al. (1995) and Abolafia and Peña-Santiago (2017a), respectively.

### Scanning electron microscopy (SEM)

For the SEM, male and female generations were first fixed in TAF and then preserved in glycerine. Glycerine preserved specimens were used for SEM observations according to the Abolafia’s (2015) proctocol. They were hydrated in distilled water, dehydrated in a graded ethanol-acetone series, critical point dried with liquid CO_2_, mounted on SEM stubs and finally coated with gold. The mounts were examined with a Zeiss Merlin microscope (5 kV) (Zeiss, Oberkochen, Germany).

### Molecular analyses

#### DNA extraction, amplification, and sequencing

DNA was extracted from pool of juveniles isolated from cadavers of *Galleria mellonella* infected with *A. saeedi* using Qiagen DNeasy® Blood and Tissue Kit (Qiagen, Hilden, Germany) (Bhat et al., 2017). Juveniles were first washed separately with Ringer’s solution followed by washing in PBS solution (Bhat et al., 2017, 2020). They were then transferred into a sterile Eppendorf tube (0.5 ml) and DNA was extracted following manufacturer’s instructions. The ITS region was amplified using the primers 18 S: 5´-TTG ATT ACG TCC CTG CCC TTT-3´ (forward) and 28 S: 5´-TTT CAC TCG CCG TTA CTA AGG-3´ (reverse) (Vrain et al., 1992). The 18 S rDNA fragment was amplified using primers NEM18SF: 5´-CGCGAATRGCTCATTACAACAGC-3´ (forward) and NEM18SR: 5´-GGGCGGTATCTGATCGCC-3´ (reverse) (Floyd et al., 2005). The flanking segment, D2-D3 regions of 28 S rDNA was amplified using primers D2F: 5´-CCTTAGTAACGGCGAGTGAAA-3´ (forward) and 536: 5´-CAGCTATCCTGAGGAAAC-3´ (reverse) (Nadler et al., 2006). The PCR master mix consisted of ddH2O 16.8 μl, 10× PCR buffer 2.5 μl, dNTP mix (10 mM each) 0.5 μl, 1 μl of each forward and reverse primers, dream taq green DNA polymerase 0.2 μl and 3 μl of DNA extract. The PCR profiles used was: 1 cycle of 94°C for 3 min followed by 40 cycles of 94°C for 30 sec, + 54°C for 30 sec for 18 S rDNA, 52°C for 30 sec for 28 S rDNA or 55°C for 30 sec for ITS rDNA, + 72°C for 60 sec, and a final extension at 72°C for 10 min. PCR was followed by electrophoresis (45 min, 100 V) of 5 μl of PCR product in a 1% TAE (Tris-acetic acid-EDTA) buffered agarose gel stained with ethidium bromide (Bhat et al., 2018; Aasha et al., 2019). All PCR-products were sequenced using ABI 3730 (48 capillary) electrophoresis instrument by Bioserve Pvt. Ltd (Hyderabad, India) and sequencing results were submitted to NCBI with accession numbers: MK935149 and MK935150 for 18 S of DH1 and KMW, respectively; MN101167 and MK935147 for 28 S of DH1 and KMW, respectively; MK935148 and MK935151 for ITS of DH1 and KMW, respectively.

### Phylogenetic analyses

The sequences were edited and compared with those already present in GenBank using the basic local alignment search tool (BLAST) of the National Centre for Biotechnology Information (NCBI) (Altschul et al., 1990). An alignment of nematode samples together with sequences of related cephalobid species was produced for the LSU (D2-D3 rDNA), SSU, and ITS rDNA sequences using default Clustal W parameters in MEGA 6.0 (Kumar et al., 2016) and optimized manually in BioEdit (Hall, 1999). Pairwise distances were computed using MEGA 6.0 (Kumar et al., 2016). All characters were treated as equally weighted and gaps as missing data. *Drilocephalobus* sp. (AY284679) for the 18 S tree and *Teratolobus* sp. (KJ652552) for the 28 S tree were used as the out-group taxa and to root the trees. ITS tree was not included because too few sequences are available in the GenBank database for their comparisons. The base substitution model was evaluated using jModeltest 0.1.1 (Posada, 2008). Phylogenetic trees were elaborated using the Bayesian inference method as implemented in the program MrBayes 3.2.7 (Ronquist et al., 2012). The HKY + Γ (gamma distribution of rate variation with a proportion of invariable sites) model was selected. The selected model was initiated with a random starting tree and run with the Markov Chain Monte Carlo for 10^6^ generations. The Bayesian tree was ultimately visualized using the FigTree program 1.4.3 (Rambaut, 2018).

## Results and discussion

The morphological and morphometrical studies and molecular (D2-D3, 18 S and ITS rDNA) analyses confirmed the present strains KMW and DH1 as conspecific to *A. saeedi* (Siddiqi et al., 1992) and hence, described as the same. This is the first report of this species from Indian subcontinent.

### Morphological characterization

*A. saeedi* (Siddiqi et al., 1992) (Figs. 1–4).

Material examined: 20 females, 21 males and 27 L3 juveniles in each KMW and DH1 populations (obtained from *Galleria* specimens from agricultural soils).

Measurements: see Tables 1 and 2.

**Table 1.** Morphometric data for *Acrobeloides saeedi* KMW isolated from *Galleria* culture.

Characters	Female	Male	Juvenile
n	20	20	27
Total body length	1387 ± 63 (1307–1566)	987 ± 89 (812–1156)	653 ± 20 (626–704)
a	14.8 ± 1.4 (12.8–17.4)	21 ± 2.7 (13.0–24.0)	22 ± 1.7 (19.6–28.0)
b	7.8 ± 0.7 (6.9–10.0)	5.9 ± 0.6 (4.7–7.0)	4.4 ± 0.2 (4.0–5.0)
c	26 ± 2.2 (22.0–33.0)	25 ± 2.7 (21.0–30.0)	17 ± 1.2 (15.0–21.0)
c’	1.8 ± 0.2 (1.5–2.3)	1.7 ± 0.2 (1.2–2.2)	2.0 ± 0.2 (1.4–2.6)
V	70 ± 2.1 (66–74)	–	–
Lip length	5.0 ± 0.6 (4–7)	4.6 ± 0.8 (3–6)	4.3 ± 0.7 (3–6)
Lip region width	8.9 ± 0.8 (8–11)	6.3 ± 0.7 (5–8)	5.0 ± 0.5 (4–5)
Stoma length	12.9 ± 2.2 (9–15)	14.6 ± 1.9 (11–17)	12 ± 1.6 (7–14)
Pharyngeal corpus length	108 ± 8.1 (88–124)	93 ± 8.9 (81–108)	82 ± 7.4 (69–99)
Isthmus length	37 ± 4.4 (28–46)	35 ± 6.0 (28–50)	29 ± 4.4 (21–37)
Basal bulb length	39 ± 4.9 (31–53)	34 ± 3.4 (28–41)	24 ± 1.5 (21–27)
Pharynx length	184 ± 11.1 (159–202)	161 ± 12.9 (142–181)	136 ± 7.8 (116–149)
Nerve ring – ant. end	113 ± 14 (91–151)	106 ± 10.2 (88–129)	94 ± 8.4 (76–109)
Excretory pore – ant. end	138 ± 11.0 (112–157)	129 ± 12.4 (112–165)	111 ± 8.9 (94–127)
Deirid – ant. end	155 ± 13.4 (130–178)	128 ± 11.9 (111–156)	–
Neck length	191 ± 9.7 (168–208)	181 ± 12.8 (156–203)	162 ± 12.4 (130–181)
Body diam. at midbody	95 ± 10.0 (80–112)	49 ± 7.7 (40–73)	30 ± 2.1 (24–33)
Ovary length	542 ± 60 (401–652)	–	–
Spermatheca length	49 ± 9.8 (33–61)	–	–
Uterus length	280 ± 46 (211–376)	–	–
Postvulval uterine sac length	100 ± 11.3 (85–112)	–	–
Vagina length	26 ± 6.2 (17–35)	–	–
Body diam. at vulval level	84 ± 5.7 (74–93)	–	–
Vulva – anterior end	976 ± 36 (896–1046)	–	–
Rectum length	24 ± 5.5 (12–32)	–	19.5 ± 2.1 (14–22)
Body diam. at anus	31 ± 3.4 (22–39)	24 ± 3.3 (18–34)	20 ± 2.7 (16–31)
Tail length	54 ± 4.1 (44–62)	39 ± 3.5 (34–48)	40 ± 2.9 (31–45)
Phasmid to anus distance	30 ± 3.4 (26–35)	28 ± 3.9 (21–36)	–
Spicules length	–	48 ± 4.2 (41–54)	–
Gubernaculum length	–	25 ± 2.8 (21–30)	–

**Notes:** All measurements are in μm (except n, ratio, and percentage) and in the form: mean ± SD (range). – = character absent.

**Table 2.** Morphometric data for *Acrobeloides saeedi* DH1 isolated from *Galleria* culture.

Characters	Female	Male	Juveniles
n	20	20	27
Total body length	1271 ± 112 (1060–1446)	959 ± 74 (798–1144)	474 ± 54 (404–636)
a	14.1 ± 1.3 (11.5–16.6)	20 ± 1.8 (17.1–24.0)	21 ± 3.5 (15.3–28.0)
b	6.9 ± 0.4 (6.1–7.8)	5.0 ± 0.3 (4.2–5.6)	3.8 ± 0.36 (3.2–4.9)
c	27 ± 2.2 (22.0–30.0)	27 ± 3.0 (22–34)	13 ± 2.3 (5.7–17.6)
c'	1.7 ± 0.2 (1.5–2.4)	1.6 ± 0.1 (1.3–1.8)	2.7 ± 0.7 (2.0–6.1)
V	71 ± 3.6 (60–77)	–	–
Lip length	4.8 ± 0.7 (3–6)	3.8 ± 0.5 (3–5)	2.8 ± 0.5 (2–4)
Lip region width	7.8 ± 1.4 (5–10)	6.3 ± 0.7 (5–8)	5.1 ± 0.7 (4–7)
Stoma length	13.1 ± 1.2 (11–16)	12.2 ± 1.6 (8–15)	10.4 ± 1.9 (8–15)
Pharyngeal corpus	107 ± 9.8 (86–125)	113 ± 6.7 (99–123)	85 ± 8.8 (64–100)
Isthmus	26 ± 4.5 (16–34)	28 ± 4.4 (21–36)	18.2 ± 4.0 (9–27)
Basal bulb length	37 ± 3.6 (30–43)	34 ± 4.1 (26–40)	21 ± 2.9 (16–30)
Pharynx length	170 ± 10.3 (155–188)	174 ± 8.4 (146–184)	122 ± 11.9 (105–142)
Nerve ring – ant. end	112 ± 6.6 (98–124)	131 ± 8.0 (114–143)	82 ± 9.1 (65–102)
Excretory pore – ant. end	134 ± 12.6 (115–161)	157 ± 12.5 (141–192)	95 ± 11.8 (77–123)
Deirid – ant. end	127 ± 17.3 (95–159)	125 ± 7.5 (105–139)	?
Neck length	184 ± 10.2 (170–202)	191 ± 8.1 (175–207)	125 ± 9.4 (108–153)
Body diam. at midbody	90 ± 10.9 (70–108)	47 ± 3.1 (40–54)	23 ± 3.4 (18–31)
Ovary length	437 ± 51 (348–532)	–	–
Spermatheca length	50 ± 10.4 (39–68)	–	–
Uterus length	385 ± 85 (256–537)	–	–
Postvulval uterine sac length	92 ± 8.6 (73–101)	–	–
Vagina length	23 ± 1.83 (19–25)	–	–
Body diam. at vulva level	75 ± 10.8 (56–91)	–	–
Vulva – anterior end	904 ± 87 (749–1043)	–	–
Rectum length	33 ± 4.8 (22–43)	–	13.4 ± 2.2 (10–18)
Body diam. at anus	28 ± 2.4 (21-32)	23 ± 2.7 (20-28)	14.3 ± 1.6 (11-17)
Tail length	48 ± 3.8 (41-54)	36 ± 2.5 (32-40)	38 ± 9.9 (29-80)
Phasmid to anus distance	27 ± 4.0 (21-37)	21 ± 2.1 (18–25)	–
Spicule length	–	45 ± 2.8 (41-50)	–
Gubernaculum length	–	26 ± 2.0 (22-30)	–

**Notes:** All measurements are in μm (except n, ratio, and percentage) and in the form: mean ± SD (range). – = character absent, ? = character not observed.

Female: Body is larger, 1.31 to 1.57 mm long, in the KMW population and smaller, 1.06 to 1.45 mm, in the DH1 population, more or less fusiform with a sudden narrowing behind the vulva, tapering anteriorly from mid-pharynx to lip region, fusiform, slightly arcuated ventrally and becomes open C shaped upon heat killing. Cuticle with annuli separated from each other by a narrow groove. Lateral fields with four alae limited by five longitudinal incisures ending at tail tip terminus, showing only three incisures after the phasmids. Lip region bears six inner labial papillae and four outer cephalic papillae. Lips are in pairs, with smooth margin; primary axils are “U”-shaped, usually with acute tip; secondary axils are “V”-shaped; guard processes are absent. Labial probolae is low, triangular in section, connected by tangential ridges. Amphidial apertures are pore like, oval. Oral opening is triangular leading into a narrow cephaloboid stoma bearing well-developed refringent rhabdia, cheilostom is short with bar-shaped cheilorhabdia, gymnostom is very short and stegostom is elongated with robust rhabdia. Pharynx is cephaloboid, divided in three regions: pharyngeal corpus is slightly fusiform, 2.7 to 3.1 times the isthmus length in KMW population while 3.7 to 5.4 times in case of DH1; isthmus is robust and basal bulb is spheroid with well-developed valvular apparatus. Excretory pore is located at isthmus level, at 60 to 89% of neck length, at 53 annuli; renette cells are just behind pharyngeal bulb. Hemizonid is present just anterior to the excretory pore. Deirids are present at basal bulb level, at 70 to 92% of neck length, at 48 annuli. Nerve ring surrounds the isthmus at metacorpus-isthmus junction or slightly posterior. Intestine with anterior end with thinner walls. Reproductive system is monodelphic, prodelphic: ovary well developed, with several lines of oocytes, with or without a double flexure at postvulval region; oviduct short; spermatheca well developed, 0.4 to 0.5 times longer than the body width; uterus is very long, divided in two parts only observed in young females, one distal tubular part and other proximal swollen part with thinner walls; in old females all length usually swollen containing 16 to 30 uterine eggs, 41 to 55 μm long and 24 to 35 μm wide; post-vulval uterine sac 0.7 to 0.9 times the body width; vagina is straight or slightly arcuate, 21 to 31% of body width; vulva ventral. Rectum is distinct, shorter than anal body width with three unicellular glands at its junction with the intestine. Anus is large, directed posteriorly. Tail is straight, conoid, truncated to slightly rounded terminus with 15 to 20 annuli ventrally. Phasmids are distinct pore like and located at 59 to 62% of tail length.

Male: Body is 0.81 to 1.16 mm long in the KMW population, and 0.80 to 1.14 mm long in the DH1 population, “J” shaped after heat killing with general morphology similar to female. Reproductive system is monorchic with testis ventrally reflexed anteriorly. Two deep latero-subventral grooves are extended from the sides of the cloacal apparatus approximately to the first preanal pair of the papillae. Genital papillae are in eight pairs, three pairs are pre-cloacal and five pairs are post-cloacal (two at mid tail length, one lateral at lateral field and one subventral, and three terminal, two subventral and one subdorsal), and one midventral papillae. Phasmids are well observed, located posterior to the anterior lateral papillae, at 67 to 70% of tail length. Spicules are long, broad and arcuate, larger than gubernaculum, with manubrium reduced, ventrally bent, rounded-elongate, calamus is conoid and lamina is slightly ventral curved with angular dorsal hump, long ventral velum and very thin rounded tip. Gubernaculum with manubrium-corpus is almost straight, well developed crura with acute tip. Tail is conoid, ventrally curved, with blunt terminus bearing a short fine mucro.

Third stage juvenile (L3): Body is robust, 0.62 to 0.70 mm long in the KMW population, and 0.40 to 0.64 mm in the DH1 population, elongate, straight or slightly curved at posterior end. Cuticle is almost smooth; lip region is similar to adult specimens. Stoma is narrow. Pharynx is clearly visible and differentiated into the three cephaloboid parts. Nerve ring surrounds the isthmus. Excretory pore is at isthmus level. Deirid is obscure. Cardia is reduced, surrounded by intestinal tissue. Rectum is 6 to 7% times the rectum width. Anus is prominent. Tail is conoid with an acute tip.

### Diagnosis (of Indian populations)

The material examined of *A. saeedi* from India is characterized by having 1.06 to 1.57 mm in females and 0.80 to 1.16 mm in males, lateral field with five longitudinal incisures, lip region with six paired lips, smooth, primary and secondary axils lacking guard processes, labial probolae low, triangular in section and frontally flattened, stoma cephaloboid with rounded cheilorhabdia, pharynx cephaloboid with slightly swollen metacorpus, female reproductive system monodelphic-prodelphic, dextral, with spermatheca well developed and postvulval uterine sac slightly shorter than the body diam., female rectum shorter than anal body diam., female tail conoid with truncate to slightly rounded terminus (41-54 μm long, c = 22.0-33.0, c’ = 1.5-2.4), male tail conoid, ventral curved (32-40 μm long, c = 21.0-34.0, c’ = 1.2-2.2), spicules 41 to 54 μm long with reduced ventral bent manubrium and slightly humped lamina, gubernaculum 21 to 30 μm long.

### Relationships

Both populations (KMW and DH1) examined now of *A. saeedi* from India agree well with the type material described by Siddiqi et al. (1992). Morphometric measurements were in close proximity to the Pakistani population described by Siddiqi et al. (1992) (Table 3).

**Table 3.** Comparative morphometrics of females from populations of *Acrobeloides maximus* – group (all measurements in μm except L in mm).

Species	*A. saeedi* (KMW-DH1)	*A. saeedi*	*A. saeedi*	*A. saeedi* (as *A. ishraqi*)	*A. saeedi* (as *A. gossypii*)	*A. saeedi* (as *A. gossypii*)	*A. bodenheimeri*	*A. bodenheimeri*	*A. bodenheimeri* (as *A. rotundifolius*)	*A. bodenheimeri*	*A. bodenheimeri*	*A. bodenheimeri*	*A. bodenheimeri*	*A. bodenheimeri* (as *A. mushtaqi*)	*A. longiuterus*	*A. longiuterus* (as *A. camberenensis*)	*A. longiuterus* (as *A. camberenensis*)	*A. longiuterus*	*A. maximus*	*Paracephalobus litoralis*
Reference	Present paper	Siddiqi et al. (1992)	Khan and Hussain (1997)	Pervez (2011)	Nahiyoon et al. (2019)	Nahiyoon et al. (2019)	Steiner (1936); Siddiqi et al. (1992)	Andrássy (1967)	Bussau (1991)	Siddiqi et al. (1992)	De Ley et al. (1999)	Abolafia and Peña-Santiago (2002)	Mehdizadeh et al. (2013)	Pervez (2011)	Rashid and Heyns (1990)	De Ley et al. (1990)	De Ley et al. (1999)	Abolafia and Peña-Santiago, 2017a, b	Thorne (1925)	Akhtar (1962)
Country	India	Pakistan	Pakistan	India	Pakistan	Pakistan	Israel	Mongolia	Denmark	Malawi	USA	Spain	Iran	India	Namibia	Senegal	USA	Namibia	USA	Pakistan
n (♀)	40	30	20	10	15	15	8	12	1	22	30	4	3	10	21	5	15	3	1	1
L	1.06–1.57	0.88–1.21	0.86–1.20	0.99–1.19	0.80–1.70	0.8–1.70	0.63–0.78	0.67–0.77	0.88	0.64–0.91	0.86–1.53	0.70–0.91	0.63–0.72	0.61–0.68	0.57–0.88	0.45–0.67	0.88–1.19	0.74–0.84	1.2	0.8
A	11.5–17.4	21.0–30.0	21.0–30.0	10.5–15.8	8.1–15.5	8.1–15.5	15.0–16.0	15.0–17.0	24.4	17–23	16.0–23.0	17.5–22.9	21.0–22.0	31.8–33.1	16.8–24.0	18.3–19.6	15.0–19.0	18.1–22.8	20.0	23.2
B	6.1–10.0	4.6–5.9	4.86–5.8	4.8–6.1	4.5–8.0	4.5–8.0	5.0–5.6	4.8–5.5	4.7	4.7–5.6	5.6–7.8	4.5–5.5	4.2–5.1	4.2–4.4	3.5–5.3	3.4–4.6	5.7–6.9	4.4–5.0	5.7	6.0
C	22.0–33.0	20.0–26.0	20–26	20.6–23	16.0–29.2	16–29.2	17–21	16.0–19.0	18.1	16.0–18.3	20.0–30.0	19.0–20.3	16.0–20.0	14.3–17.9	16.2–21.8	15.1–18.0	20.0–26.0	16.5–19.6	18.0	17.7
c'	1.5–2.4	1.8–2.4	1.8–2.3	1.4–1.8	1.1–1.9	1.1–1.9	?	1.7–2.1	2.1*	2.0–2.5	1.4–2.1	1.6–2.1	1.8–2.1	1.2–1.8	1.4–2.3	2.0–2.4	1.4–1.9	1.7–2.1	2.0	2.5–2.7*
V	60–77	65–74	?	68–74	70–75	70.2–75	69–71	65–71	69	67–71	67–71	64–69	67–70	77*	64–72	65–70	84*	66–70	70	65
Lip region width	5–11	11–13	11–13	11–13	9–10	12–15	9–10	?	6.7*	5.9*	9*	3	10–11	8.6–12	?	?	?	5.5*	?	7*
Stoma length	9–18	12*	12–18	16–16	12–14	12–22	12–14	?	20*	14.7*	11–16	11–14	13–14	8–12	11–14	8–10	12–15	10–12	20	10*
Procorpus	37–69	?	?	?	?	?	?	?	?	?	50–79	?	?	?	?	?	60–85	?	?	?
Metacorpus	43–80	?	?	?	?	?	?	?	?	?	30–49	?	?	?	?	?	35–46	?	?	?
Isthmus	16–46	21.9*	20*	?	20–26	14–28	20–26	?	23*	22–36	20–37	22–25	21–25	27.6*	14–15*	15–24	17–23	15 –23	?	?
Bulb length	30–53	29–34	29–34	24–38	19–21	26–32.5	19–21	?	19.8*	19–21	22–32	22–27	19–26	24*	?	15–21	22–28	24–25	?	13–25
Pharynx length	155–202	190–218	190–218	179–223	129–157	166–228	129–157	?	157*	135–178	205–316*	135–166	133–141	145–154	?	123 –159	229–313*	137–164	210	8–14
Nerve ring–ant. end	91–151	163*	?	151*	92–114	125–136	92–114	?	132*	106*	113–174	107–126	109–117	101–105	89–142	82–108	125–148	119–135	162	78*
Excretory pore–ant. end	112–161	140–188	140–188	155–174	?	136–150	?	?	139*	118–134	131–209	114–148	123–135	?	89–157	83–118	154–195	127–142	?	81*
Neck region	168–208	215*	202–236*	194–239*	?	178–250*	?	?	177*	137*	157–332*	146–180	139–150	153–166*	120–177	134–153	241–327*	149–172	?	?
Midbody diam.	70–112	56.6*	?	59–113	?	52–170	?	?	40*	39*	49–73	33–40	30–34	18–21	?	13–17	49–72	35–41	?	29.4*
Vulva – anterior end	749–1046	680–840	573*	637*	503–550	616–1225	503–550	?	650*	503–550	1001*	447–629	436–491	495.2*	?	?	875*	?	840	291.6*
Body diam. at anus	21–39	29*	19*	28–36	?	25–50	?	?	30*	18*	22–31	18–22	19–20	19–21	?	20–23	25*	21–25	34	15*
Rectum length	12–43	37.5*	?	31–42	?	24–30	?	?	?	19*	27–42	22–25	21–29	17.2*	?	?	23–32	17.8	?	?
Tail length	41–62	39–50	39–50	10–15	35–45	42–55	35–45	?	50*	39–56	41–60	36–45	35–40	37–42	61–111	30–37	41–52	88–91	66	39–43
Phasmid to anus distance	21–39	10–19	23.3*	?	?	?	?	?	?	18.8*	22–36	16–29	17.5*	20.7*	41–66	?	21–32	56–64	?	?
D% (EP/ES×100)	64–101	74–86*	74–86*	82*	79*	79*	?	?	88*	80.5*	64–66	87*	94*	?	?	?	68*	88*	?	?
E% (EP/T×100)	206–320	359–376*	359–376*	?	?	285*	?	?	278*	265.3*	312–348	323*	344*	?	?	?	378*	150*	?	?

**Notes:** References (Ref.): 1– Present paper, 2– Abolafia and Peña-Santiago (2002), 3– Abolafia and Peña-Santiago (2017b), 4– Akhtar (1962), 5– Andrássy (1967), 6– Bussau (1991), 7– De Ley et al. (1990), 8– De Ley et al. (1999), 9– Khan and Hussain (1997), 10– Mehdizadeh et al. (2013), 11– Nahiyoon et al. (2019), 12– Pervez (2011), 13– Rashid and Heyns (1990), 14– Siddiqi et al. (1992), 15– Steiner (1936), 16– Thorne (1925). * = measurements from drawings, ? = measurement unknown.

Additionally, *A. saeedi* resembles morphologically with *A. bodenheimeri* (Steiner, 1936; Thorne, 1937), *A. longiuterus*, and *A. maximus* (Tables 3 and 4). However, from *A. bodenheimeri*, the Indian populations can be distinguished on the basis of the position of the uterus with respect to the intestine which is dextral (right-handed) in present strains (KMW and DH1) and sinistral (left-handed) in *A. bodenheimeri*; postvulval uterine sac with shorter range (85-112 vs 45-132 μm), female body length with less range (1.03-1.57 vs 0.87-1.53 mm); pharyngeal basal bulb longer (31-53 vs 22-32 μm), nerve ring to anterior end more anterior (91-151 vs 113-174 μm), distance from anterior end to excretory pore shorter (112-157 vs 131-209 μm), distance from anterior end to deirid shorter (130-178 vs 212 μm), rectum shorter (12-32 vs 27-42 μm).

**Table 4.** Comparative morphometrics of males from populations of *Acrobeloides maximus* – group (all measurements in μm except L in mm).

Species	*A. saeedi* (KMW-DH1)	*A. longiuterus*	*A. longiuterus* (as camberenensis)	*A. longiuterus* (as A. camberenensis)	*A. longiuterus*	*A. saeedi* (as *A. gossypii*)	*A. saeedi* (as *A. gossypii*)	*A. bodenheimeri*	*A. bodenheimeri*	*A. bodenheimeri*	*A. bodenheimeri*	*A. bodenheimeri*	*A. bodenheimeri*	*A. bodenheimeri* (as A. mushtaqi)	A. maximus
Reference	Present paper	Rashid and Heyns (1990)	De Ley et al. (1990)	De Ley et al. (1999)	Abolafia and Peña-Santiago, 2017a, b	Nahiyoon et al. (2019)	Nahiyoon et al. (2019)	Steiner (1936); Siddiqi et al. (1992)	Andrássy (1967)	Siddiqi et al. (1992)	De Ley et al. (1999)	Abolafia, Peña-Santiago (2002)	Mehdizadeh et al. (2013)	Pervez (2011)	Steiner (1935)
Country (State)	India	Namibia	Senegal	USA	Namibia	Pakistan	Pakistan	Israel	Mongolia	Malawi	USA	Spain	Iran	India	USA
n (♂)	40	26	6	20	6	15	15	8	6	10	10	2	4	10	1
L	0.79–1.16	0.53–0.94	0.54–0.65	0.70–1.03	0.68–0.84	0.71–1.39	0.71–1.39	0.63–0.71	0.56–0.59	0.56–0.87	0.98–1.18	0.69, 0.73	0.70–0.86	0.61–0.62	0.9
a	13.0–24.0	16.7–26.4	20.2–21.7	16.0–26.0	17.8–23.7	11.1–16.6	11.1–16.6	14.0–16.0	12.0–15.0	22–26	20.0–25.0	24.0, 25.0	22.0–30.0	27.0–31.0	27.0
b	4.2–7.0	4.0–5.9	4.0–4.8	4.3–6.8	4.1–5.1	4.6–7.5	4.6–7.5	4.9–5.1	4.2–4.8	4.3–5.4	6.0–7.8	5.0, 5.2	4.8–5.4	4.0–4.2	7.2
c	21.0–34.0	14.4–21.7	14.9–18.0	17.0–23.0	17.3–20.3	19.5–27.8	19.5–27.8	15–18	14.0–16.0	15–18	20.0–24.0	18.8, 16.6	14.0–18.0	16.0–18.0	18.0
c’	1.2–2.2	1.4–2.1	1.6–2.0	1.2–1.6	1.2–1.5	0.9–1.3	0.9–1.3	?	1.9*	2.1*	1.3–1.7	1.3, 1.7	1.6–2.0	1.4–1.8	1.8*
Lip region width	5–8	?	?	?	?	11–14	11–14	?	?	?	?	3, 4	3–4	?	?
Stoma length	8–17	?	08–10	11–13	11–12	12–15	12–15	?	?	?	10–14	11, 12	10–11	11–12	?
Procorpus length	28–59	?	?	58–66	?	?	?	?	?	?	?	11, 11	12–14	?	?
Metacorpus length	45–75	?	?	32–40	?	?	?	?	?	?	?	?	?	?	?
Isthmus length	21–50	?	15–25	16–23	19–22	16–20	16–20	22–23	?	?	?	?	17–32	?	?
Bulb length	26–41	?	16–23	17–27	22–28	24–30	24–30	?	?	?	22–30	22, 23	20–26	?	?
Pharynx length	142–184	?	122–151*	205–260	122–161	150–188	150–188	129–143	?	?	210–293	100, 119	131–149	145–152	?
Nerve ring – ant. End	88–143	?	90–104	108–135	120–132	112–134	112–134	100–115	?	?	117–151	140, 140	110–127	106–110	?
Excretory pore – ant end	112–192	?	78–91	121–175	119–141	132–144	132–144	?	?	?	135–192	121, ?	128–143	?	?
Neck length	156–207	130–174	128–148	?	156–168	162–203*	162–203*	?	?	?	133–188		143–160	?	?
Midbody diam.	40–73	?	25–31	39–51	35–42	64–88	64–88	?	?	?	44–57	33, 40	24–40	19–22	?
Anal body diam.	18–34	?	20–23	27–35	27–34	32–40	32–40	?	?	25*	31–35	18, 22	24–27	17–24	?
Tail length	32–48	37–50	36–42	39–47	38–44	40–50	40–50	38–43	?	54–80	40–58	22, 25	43–54	34–37	?
Spicules length	41–54	29–51	29–34	40–48	41–46	38–57	38–57	39–44	30–40	35–43	42–50	37	38–41	39–43	?
Gubernaculum length	21–30	18–35	14–19	24–30	26–31	24–35	24–35	22–28	20	19–24	27–34	23, 22	19–28	23–24	?
D% (EP/ES×100)	69–94	?	?	57–67*	92*	82*	82*	?	?	?	?		97*	?	?
E% (EP/T×100)	242–577	?	?	310–372*	317*	306*	306*	?	?	?	?	233, 300*	279*	?	?
SW% (SL/ABD×100)	150–294	?	?	151*	143*	126*	126*	?	?	?	?	137*	155*	194*	?
GS% (GL/SL×100)	40–70	?	?	67*	66*	55*	55*	?	?	?	?	65*	59*	58*	?

**Notes:** References (Ref.): 1– Present paper, 2– Abolafia and Peña-Santiago (2002), 3– Abolafia and Peña-Santiago (2017b), 4– Andrássy (1967), 5– De Ley et al. (1990), 6– De Ley et al. (1999), 7– Mehdizadeh et al. (2013), 8– Nahiyoon et al. (2019), 9– Pervez (2011), 10– Rashid and Heyns (1990), 11– Siddiqi et al. (1992), 12– Steiner (1935), 13– Steiner (1936). * = measurements from drawings, ? = measurement unknown.

From *A. longiuterus* described by Rashid and Heyns (1990) (redescribed by Abolafia and Peña-Santiago, 2017b, authors who synonymized it with *A. camberenensis* described by De Ley et al., 1990, 1999, its junior synonym), it can be distinguished by having longer body size of females (1.31-1.57 vs 0.65-0.86 mm), neck comparatively longer (168-208 vs 135-175 μm), longer isthmus (28-46 vs 14.5-19 μm), shorter phasmid to anus distance (26-39 vs 49-65 μm, longer tail (44-62 vs 37-45 μm), longer postvulval uterine sac (85-112 vs 75-101 μm) and Demanian indices. Males can be distinguished by longer size (0.81-1.16 vs 0.61-0.89 mm), comparatively longer neck (156-203 vs 143-171 μm), *b* ratio (4.7-7.0 vs 4.1-5.5 μm), *c* ratio (21-30 vs 16-21 μm), stoma (11-17 vs 11-12 μm), isthmus (28-50 vs 19-22 μm), nerve ring (28–41 vs 22–28 μm), neck size (156-203 vs 156-168 μm), mid-body diam. (40-73 vs 35-42 μm) and excretory pore position (112–165 vs 119-145 μm); while some measurements like pharyngeal corpus (81-108 vs 101-111 μm), nerve ring (88-129 vs 120-132 μm) and phasmid to anus (21-36 vs 50-64 μm) were comparatively shorter.

From *A. maximus*, Indian strains (KMW and DH1) can be distinguished by having lips lacking seta-like processes (vs bearing seta-like process at primary axils), pharyngeal metacorpus slightly fusiform (vs fusiform in Thorne (1925) but not well appreciated in Steiner (1936)), lateral field with five incisures (vs three according Smythe and Nadler (2006), being unknown in Thorne (1925) and Steiner (1936)), males as frequent as females (vs male rare or absent, presumably parthenogenetic females (Smythe and Nadler, 2006)), female tail terminus truncate (vs finely rounded). Although the size of the females of the Indian populations of *A. saeedi* are similar to *A. maximus* (1.31-1.57 (1.2-1.4) vs 1.2 mm) but they differed in Demanian indices.

### Molecular characterization and its taxonomical implications

*A. saeedi* strains DH1 and KMW were molecularly characterized by ITS rDNA (901 bp, 938 bp), 18 S rDNA (894 bp, 895 bp) and flanking regions D2-D3 of rDNA (984 bp, 997 bp), respectively. The NBlast analysis of D2-D3, 18 S and ITS rDNA sequences of present specimens showed 100% similarity with D2-D3 (KY914573), 18 S (KY090631) and ITS (KY090632) rDNA sequences of *Acrobeloides* sp. ES-2017 isolate SMF3 from Iran. 18 S sequences of the present two strains do not show any nucleotide difference with each other and with *Acrobeloides* sp. ES-2017 present in the GenBank. ITS and D2-D3 sequences of DH1 do not show any nucleotide difference with *Acrobeloides* sp. ES-2017 (KY090632), however, together these regions show two and one nucleotide differences with KMW, respectively. According to this, the *Acrobeloides* material from Iran could be considered conspecific with *A. saeedi*.

On the other hand, *A. saeedi* was considered a probable junior synonym of *A. maximus* by De Ley et al. (1999) based on morphological data. However, the 18 S sequence alignment of present strains DH1 and KMW showed 21 bp differences with *A. maximus* (JQ237850), while 28 S sequence alignment showed 51 bp differences and three gaps with *A. maximus* (AF147067). ITS sequences of *A. maximus* are lacking. This shows that both species are not conspecific.

On the other hand also, *A. saeedi* displays some similar morphology with *A. longiuterus*, two almost undistinguished taxa. However, molecularly both are different. Our D2-D3 sequences of *A. saeedi* when aligned with only one available D2-D3 sequence (AF147069) of *A. longiuterus* (formerly *A. camberenensis*), it showed 38 bp differences. Also, alignment of ITS rDNA of present two strains DH1 and KMW with ITS rDNA of *A. longiuterus* (MG946132) from Sri Lanka showed 73 bp differences and 23 gaps. According to this, both taxa must be maintained separated.

With respect to *A. bodenheimeri* (AF202162), the sequence alignment of 18 S genes of present strains showed 22 bp differences. In the D2D3 expansion fragment of 28 S genes, 54 bp differences were observed in aligned data of present strains with DQ145625 (*A. bodenheimeri*) from USA. These confirm the present strains to be different from *A. bodenheimeri*.

Distance matrix analyses with other closely related populations of several *Acrobeloides* species were also carried out using above three genes studies. Thus, the 18 S rDNA sequences of DH1 and KMW are separated from those of other closely related species of *Acrobeloides* by 9 to 89 bp (Table 5). The D2-D3 segment of 28 S rDNA gene in the Indian isolates differed in 5 to 76 bp from other closely related species of *Acrobeloides* (Table 6).

**Table 5.** Pairwise distances of the 18 S rDNA regions between present strains of *Acrobeloides* and already described species.

S. No.	18S rDNA	Country	1	2	3	4	5	6	7	8	9	10	11
1	MK935150 *A. saeedi* KMW	India		*0*	*0*	*9*	*19*	*19*	*19*	*19*	*24*	*30*	*89*
2	MK935149 *A. saeedi* DH1	India	*100*		*0*	*9*	*19*	*19*	*19*	*19*	*24*	*30*	*89*
3	KY090631 *A. saeedi*	Iran	*100*	*100*		9	19	19	19	19	31	23	89
4	MK541681 *A. tricornis*	Germany	*98.5*	*98.5*	98.3		0	0	0	0	10	10	9
5	DQ102707 *A. nanus*	UK	*98.5*	*98.5*	98.5	100		16	2	0	39	13	97
6	KX889085 *A. varius*	South Korea	*98.5*	*98.5*	98.5	100	99.3		2	0	39	13	96
7	AY284673 *A. apiculatus*	Netherlands	*98.5*	*98.5*	98.5	100	99.9	99.9		0	37	13	95
8	MF325099 *A. buchneri*	Germany	*98.4*	*98.4*	98.5	100	100	100	100		19	4	86
9	AF202159 *A. bodenheimeri*	France	*98.1*	*98.1*	97.4	98.6	98.3	98.3	98.4	98.4		32	92
10	KY119635 *A. thornei*	Ireland	*97.5*	*97.5*	98.1	98.2	99.0	99.0	99.0	99.7	97.4		95
11	JQ237850 *A. maximus*	USA	*92.3*	*92.3*	91.7	98.8	95.0	95.1	95.1	92.0	95.3	91.3	

**Notes:** Data of present strains shown in italic. Below diagonal, percentage similarity; above diagonal, total character difference.

**Table 6.** Pairwise distances of the D2D3 regions of 28 S rDNA regions between present strains of *Acrobeloides* and already described species.

S. No.	28S rDNA	Country	1	2	3	4	5	6	7	8	9	10	11	12	13	14
1	MK935147 *A. saeedi* KMW	India		*1*	*1*	*5*	*5*	*5*	*38*	*51*	*59*	*67*	*74*	*75*	*76*	*76*
2	MN101167 *A. saeedi* DH1	India	*100*		*0*	*5*	*5*	*5*	*38*	*51*	*59*	*68*	*74*	*75*	*77*	*77*
3	KY914573 *A. saeedi*	Iran	*100*	*100*		10	10	10	38	56	64	68	79	80	82	82
4	MF325168 *A. sexlineatus*	Germany	*98.9*	*98.9*	98.0		0	0	5	8	0	11	0	0	0	0
5	MF325157 *A. buchneri*	Germany	*98.9*	*98.9*	98.0	100		0	5	8	0	11	0	0	0	0
6	DQ903087 *A. tricornis*	Germany	*95.1*	*95.0*	94.7	100	100		5	8	0	11	0	0	0	0
7	AF147069 *A. longiuterus*	USA	*96.7*	*96.7*	96.8	99.0	99.0	96.9		30	35	45	34	35	36	36
8	AF147067 *A. maximus*	USA	*95.6*	*95.5*	95.2	98.5	98.5	96.1	97.5		43	51	44	45	46	46
9	KX889089 *A. varius*	South Korea	*94.6*	*94.6*	94.3	100	100	99.8	97.0	96.3		41	1	0	3	3
10	DQ145625 *A. bodenheimeri*	Belgium	*95.8*	*95.6*	95.7	97.7	97.7	96.9	96.1	95.6	96.4		50	49	50	50
11	DQ903076 *A. nanus*	Sweden	*95.2*	*95.2*	94.9	100	100	99.9	97.1	96.3	99.9	96.9		1	2	2
12	DQ903083 *A. thornei*	USA	*95.1*	*95.1*	94.9	100	100	99.8	97.0	96.2	100	96.9	99.9		3	3
13	DQ145624 *A. ellesmerensis*	USA	*95.1*	*95.0*	94.8	100	100	100	96.9	96.1	99.8	96.9	99.9	99.8		0
14	DQ903081 *A. buetschlii*	USA	*95.1*	*95.0*	94.8	100	100	100	96.9	96.1	99.8	96.9	99.9	99.8	100	

**Notes:** Data of present strains shown in italic. Below diagonal, percentage similarity; above diagonal, total character difference.

All of these data showed that *A. saeedi* is molecularly different with respect to its more similar species, *A. bodenheimeri*, *A. longiuterus*, and *A. maximus*, and hence, it should be considered as valid species.

### Phylogenetic analysis

**Figure 5:**
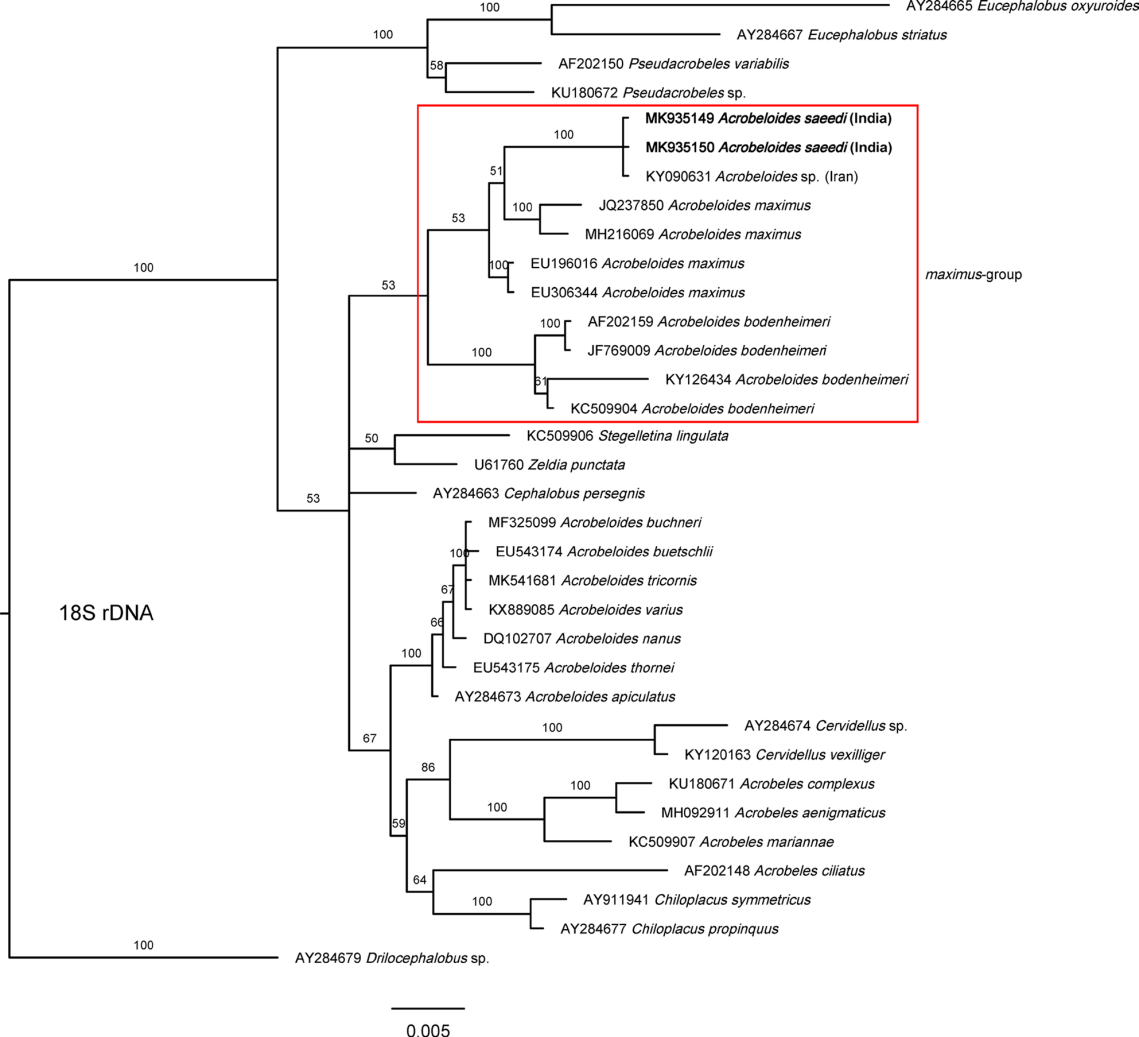
Bayesian Inference tree from known and the newly sequenced *Acrobeloides saeedi* based on sequences of the 18 S rDNA region. Bayesian posterior probabilities (%) are given for each clade. Scale bar shows the number of substitutions per site.

**Figure 6:**
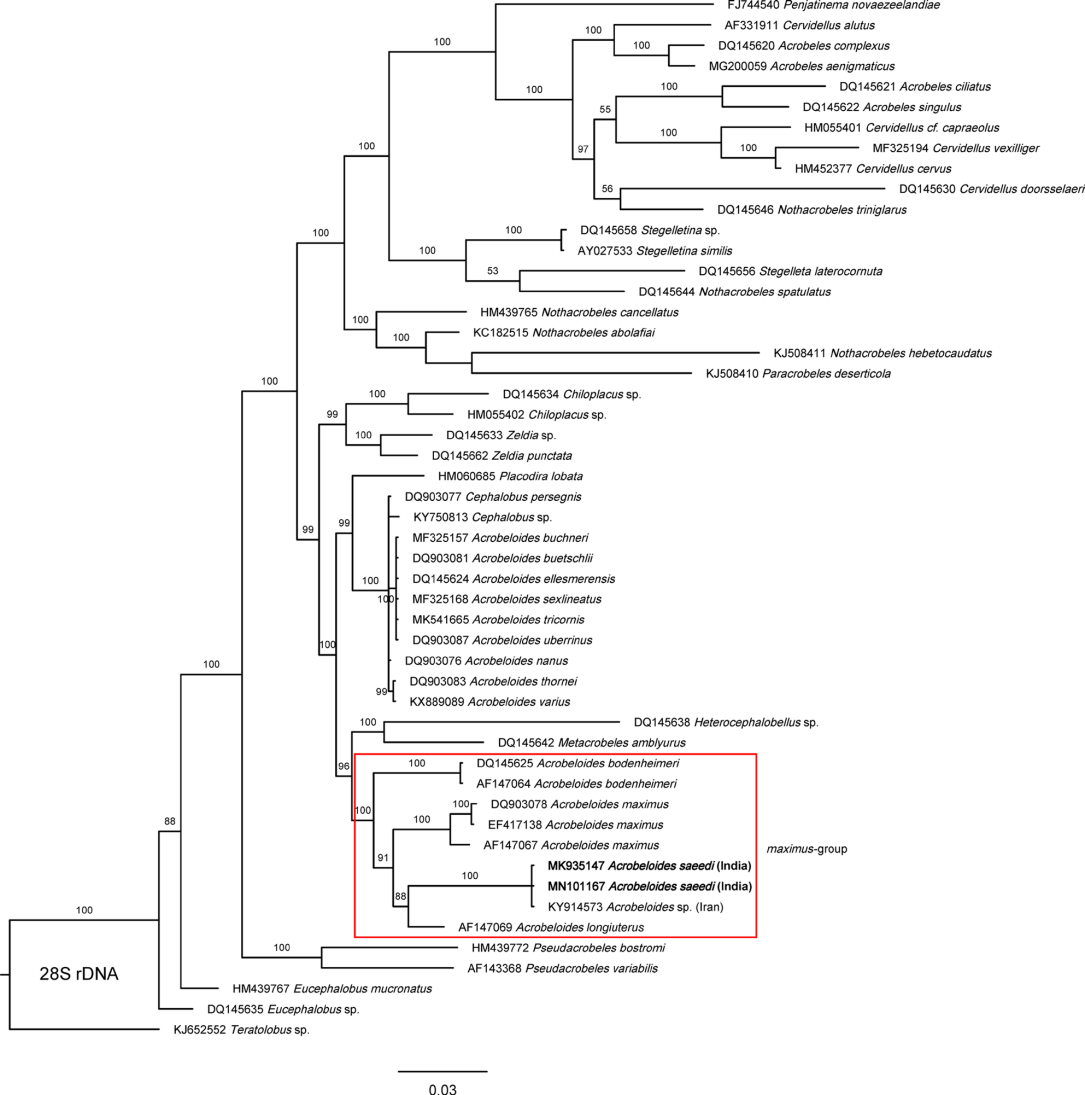
Bayesian Inference tree from known and the newly sequenced *Acrobeloides saeedi* based on sequences of the 28 S rDNA region. Bayesian posterior probabilities (%) are given for each clade. Scale bar shows the number of substitutions per site.

The phylogenetic analyses of the present stains based on 18 S rDNA and flanking region D2-D3 segment of 28 S rDNA gene also supported the molecular data. Phylogenetic analyses based on 18 S rDNA sequences (Fig. 5) showed a clear monophyly of the group formed by the isolates DH1 and KMW and other undescribed *Acrobeloides* species ES-2017 from Iran, probably conspecific isolates within a highly supported (100%) clade and together formed a sister clade with other species of “*maximus*” group from different geographical regions, namely *A. maximus* and *A. bodenheimeri*. In D2-D3 rDNA tree (Fig. 6), present two strains DH1 and KMW formed a monophyletic group with *Acrobeloides* sp. ES-2017, and together formed sister clad with *A. longiuterus* (including *A. camberenensis*, its junior synonym (Abolafia and Peña-Santiago, 2017a, b) from USA. Here also, this pair was sister to the other two species of “*maximus*” group from different geographical regions, namely *A. maximus* and *A. bodenheimeri*. For the ITS rDNA region, there were not enough sequences within *Acrobeloides* genus to construct any useful phylogenetic tree or use it for comparisons. However, both resulting sequences were added to GenBank with accession numbers of KU721840 (KMW) and KU721841 (DH1) for future comparisons.

### Taxonomical remarks

*Acrobeloides* strains DH1 and KMW obtained during the present study were conspecific to *A. saeedi* from Pakistan. Although they shared morphological similarities with *A. longiuterus*, *A. maximus* and *A. bodenheimeri* but some divergences were also found and displayed morphometrical differences (Tables 3 and 4). This is the first molecular study of this species and first valid report from India. ITS, 18 S, and D2-D3 rDNA studies confirm it to be different from morphologically closely related species of *Acrobeloides*. Molecular and phylogenetic studies based on the above three genes revealed the specimens studied now and the *Acrobeloides* population examined from Iran, could be conspecific.

On the other hand, Pervez (2011) described *A. ishraqi* as a new species from Uttar Pradesh, India. This author compared the specimens with *A. bodenheimeri* and *A. arenicola*, but did not compare it with its more similar species, *A. saeedi*, having identical morphology and morphometry. According to this, we considered both species as conspecific being *A. ishraqi* a junior synonym of *A. saeedi.*

Another species, described by Pervez (2011), *A. mushtaqi* (Pervez, 2011), was described from Uttar Pradesh, India. The author compared it with *A. bodenheimeri* and did not find very strong diagnostic characters to differentiate between them. However, their material does not have any important differences with respect to *A. bodenheimeri*. Although this author does not mention the position of the uterus with respect to the intestine (dextral or sinistral), the main character to distinguish *A. bodenheimeri* from other similar species, its morphology and morphometry agree with it and we considered *A. mushtaqi* as junior synonym of *A. bodenheimeri.*

Recently, Nahiyoon et al. (2019) described a new species, *A. gossypii* (Nahiyoon et al., 2019), from Pakistan. These authors described it using only morphological approaches and related their specimens only with *A. bodenheimeri*, but they did not compare it with its more similar species, *A. saeedi*, which has almost identical morphology and morphometry. Accordingly, we considered both species as conspecific being *A. gossypii* a junior synonym of *A. saeedi*.
